# Acute Respiratory Failure due to Neuromyelitis Optica Treated Successfully with Plasmapheresis

**DOI:** 10.1155/2016/1287690

**Published:** 2016-02-17

**Authors:** Massa Zantah, Timothy B. Coyle, Debapriya Datta

**Affiliations:** ^1^Department of Medicine, University of CT Health Center, Farmington, CT 06030, USA; ^2^Division of Pulmonary & Critical Care Medicine, University of CT Health Center, Farmington, CT 06030, USA

## Abstract

Neuromyelitis Optica (NMO) is a demyelinating autoimmune disease involving the central nervous system. Acute respiratory failure from cervical myelitis due to NMO is known to occur but is uncommon in monophasic disease and is treated with high dose steroids. We report a case of a patient with NMO who developed acute respiratory failure related to cervical spinal cord involvement, refractory to pulse dose steroid therapy, which resolved with plasmapheresis.

## 1. Introduction

Neuromyelitis Optica (NMO) is a severe demyelinating autoimmune disease involving the central nervous system. Acute respiratory failure (ARF) due to monophasic NMO is uncommon. We report a case of NMO who developed ARF related to cervical myelitis, which was refractory to pulse dose steroid therapy, but resolved with plasmapheresis.

## 2. Case Report

A 53-year-old female with a history of NMO with positive NMO-immunoglobulin G (NMO-IgG)/aquaporin-4 antibodies (AQP4-Ab) presented to the emergency department (ED) with complaints of headache, left sided weakness, numbness, and tingling for 3 days. Her medications included prednisone 5 mg daily, escitalopram, amitriptyline, and azathioprine. The patient denied smoking, alcohol, or illicit drug use.

**Figure 1:**
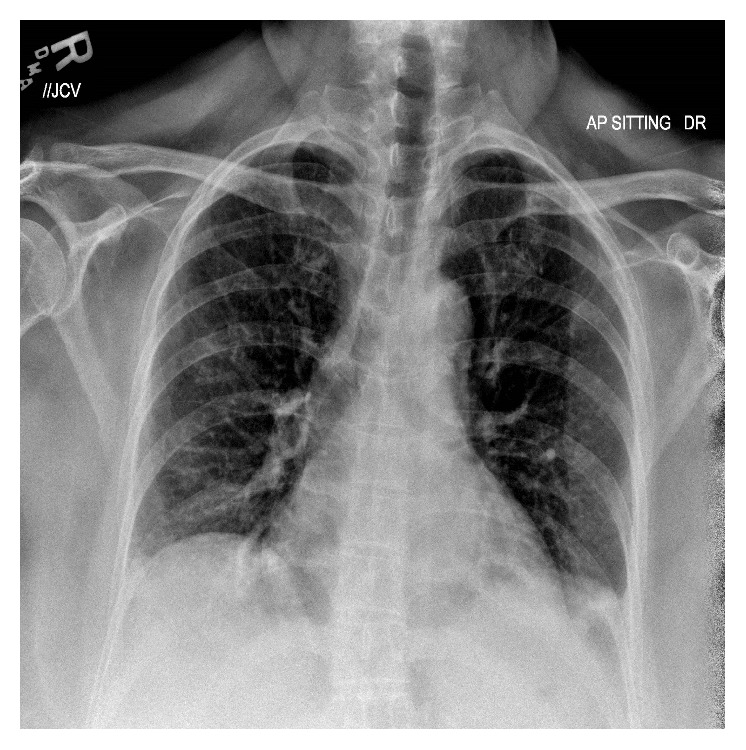
Chest X-ray showing hypoinflated lung fields, with basal atelectasis and elevated hemidiaphragms.

**Figure 2:**
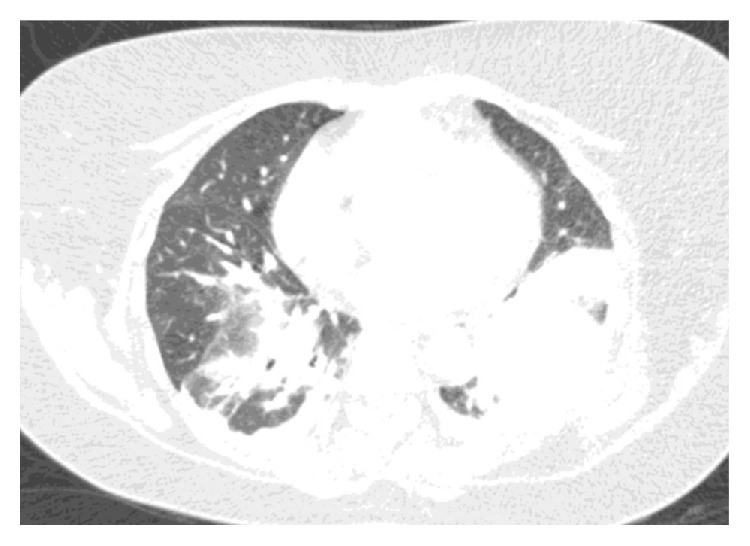
CT chest showing bibasal atelectasis, more prominent on the left.

**Figure 3:**
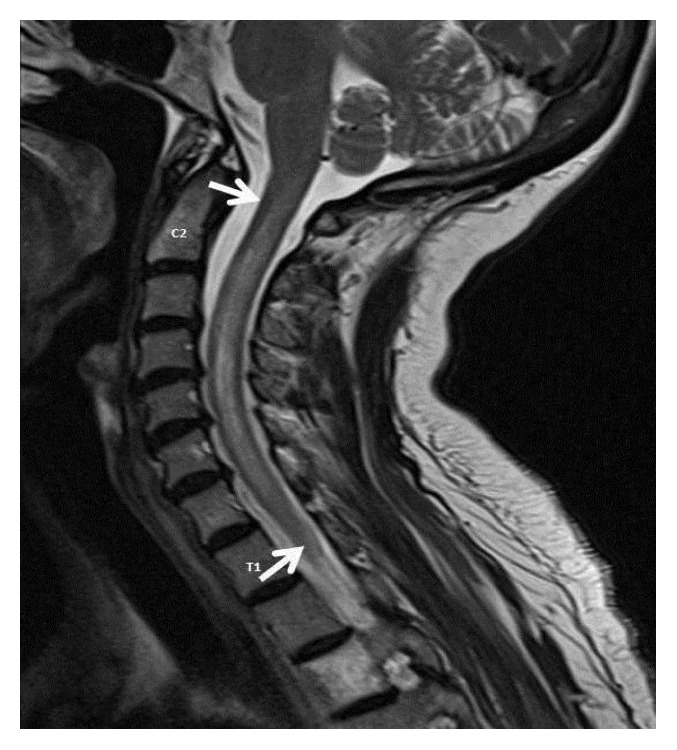
MRI of spine showing intramedullary demyelination in the spinal cord, evident as enhanced T2 signal, with peripheral contrast enhancement, extending from C2 to T1 vertebral body (arrows).

In the ED, she was in mild respiratory distress with oxygen (O_2_) saturation of 85% on room air. Other vital signs were normal. Lung and cardiovascular examination was normal. Neurological examination revealed mild weakness, decreased sensation to light touch, hyperreflexia, and a positive Babinski sign on the left. Laboratory data were normal. ABG revealed a pH of 7.46, pCO_2_ of 37.1, and pO_2_ of 60.6 on O_2_ at 2 liters/minute by nasal cannula. Chest X-ray (Figure 1) showed hypoinflated lung fields with bibasal atelectasis and elevated hemidiaphragms. The patient was started on O_2_ and intravenous pulse dose steroids and continued on her azathioprine home dose. Over the next few hours, the patient became increasingly dyspneic and hypoxic. Negative inspiratory force was −20 cm H_2_O. A computed tomogram (CT) scan of the chest (Figure 2) showed bibasilar atelectasis. Lung ultrasound revealed impaired movement of the left diaphragm, consistent with paresis. The patient was started on bilevel positive airway pressure (BIPAP). Cervical spine MRI (Figure 3) showed increased T2 signal within the spinal cord with heterogeneous enhancement in the cord extending between the mid-C2 and T1 vertebrae.

The patient's ARF was felt to be due to cervical cord involvement by NMO, resulting in diaphragmatic weakness. Patient was started on pulsed steroids: methylprednisolone at 1 gram IV daily for 3 days, followed by 1.5 gm/kg/day for 3 days, following which it was rapidly tapered off. Plasmapheresis was started on day 4 after no significant clinical response was seen after 3 days of pulsed steroid therapy. Plasmapheresis was performed daily for 5 days, following which the patient's respiratory distress and oxygenation improved and BIPAP was discontinued. Her diaphragmatic excursion normalized on fluoroscopy. Her neurologic symptoms also improved significantly.

## 3. Discussion

NMO, also known as Devic's disease, is a rare but severe inflammatory, demyelinating, and necrotizing autoimmune disease of the central nervous system which is distinct from multiple sclerosis (MS) [1]. It is characterized by recurrent attacks of optic neuritis, myelitis, and presence of NMO-immunoglobulin G (NMO-IgG)/aquaporin-4 antibodies (AQP4-Ab) [1]. The clinical manifestations of NMO are more severe than those of “typical” MS [1, 2]. ARF is unknown in MS. Cerebrospinal fluid and MRI findings can distinguish NMO from MS [3].

A revised set of criteria for diagnosis of NMO were proposed in 2006 [1]. These guidelines require two definite criteria: (i) optic neuritis and (ii) acute myelitis plus at least two of three supportive criteria: (i) contiguous spinal cord MRI lesion extending over 3 vertebral segments, (ii) brain MRI not meeting criteria for multiple sclerosis, and (iii) NMO-IgG seropositive status.

Spinal cord involvement in NMO usually presents as transverse myelitis with paraparesis or quadriparesis, a sensory level and sphincter dysfunction [4, 5]. NMO may also present as radicular pain, paroxysmal tonic spasms, nausea, and intractable hiccups [4]. Due to involvement of the respiratory center in the medulla, neurogenic ARF and death can occur [2, 3].

In one series [6], respiratory dysfunction was reported in 22% patients after onset of NMO. In 16% of these patients, respiratory failure was related to a relapse; 7% required invasive mechanical ventilation. In another series [4], ARF caused by acute cervical myelitis occurred 19 times (33%) in 16 relapsing patients and was responsible for death in 15 (93%) of these patient. Only two patients with monophasic NMO (2%) had this complication, with both patients recovering [3].

The guidelines regarding management are based on expert opinions [2, 4]. In one series [4], corticosteroids resulted in improvement in 80% of patients. Plasmapheresis resulted in improvement in 2/3rd of steroid-refractory patients. Majority of patients with acute NMO respond within 1–5 days of high dose intravenous methylprednisolone 1 gram daily for 3–5 consecutive days, followed by slow taper [2, 4]. Plasmapheresis and/or intravenous immunoglobulins are used in steroid-refractory cases [7]. Diaphragmatic pacing has also been reported to successfully wean patient with ARF due to NMO requiring mechanical ventilation [8].

In contrast to MS, maintenance therapy with immunosuppressives rather than immunomodulators achieves better prevention of recurrences [9]. Azathioprine, low dose prednisolone, or rituximab may be used for maintenance [10]. Methotrexate, cyclophosphamide, mycophenolate, and mitoxantrone have been used with variable success [4, 9].

The patient was subsequently started on rituximab. On subsequent outpatient follow-up, resolution of her neurologic symptoms was noted.
